# Supplementary material for: Androgen drives melanoma invasiveness and metastatic spread by inducing tumorigenic fucosylation
Source: Nat Commun. 2024 Feb 7;15:1148. doi: 10.1038/s41467-024-45324-w (PMC10850104; doi:10.1038/s41467-024-45324-w)
Supplement: Supplementary file 1 — Supplementary Information [file 41467_2024_45324_MOESM1_ESM.pdf]

ANDROGEN DRIVES MELANOMA INVASIVENESS AND METASTATIC SPREAD BY INDUCING TUMORIGENIC FUCOSYLATION

Qian Liu[1,2,3], Emma Adhikari[1,2,3], Daniel K. Lester[1,2,3], Bin Fang[4], Joseph Johnson[5], Yijun Tian[1], Andrea T. Mockabee-Macias[1,3], Victoria Izumi[4], Kelly M. Guzman[5], Michael G. White[7], John M. Koomen[4,8], Jennifer A. Wargo[7,9], Jane Messina[10], Jianfei Qi[11], and Eric K. Lau[1,3*]

[1]Department of Tumor Biology, H. Lee Moffitt Cancer Center & Research Institute, Tampa, Florida, USA

[2]Cancer Biology Ph.D. Program, University of South Florida, Tampa, Florida, USA

[3]Molecular Medicine Program, H. Lee Moffitt Cancer Center & Research Institute, Tampa, Florida, USA

[4]Proteomics and Metabolomics Core, H. Lee Moffitt Cancer Center & Research Institute, Tampa, Florida, USA

[5]Department of Analytic Microscopy, H. Lee Moffitt Cancer Center & Research Institute, Tampa, Florida, USA

[7]Department of Surgical Oncology, MD Anderson Cancer Center, Houston, Texas, USA

[8]Department of Molecular Oncology, H. Lee Moffitt Cancer Center & Research Institute, Tampa, Florida, USA

[9]Department of Genomic Medicine, MD Anderson Cancer Center, Houston, Texas, USA

[10]Department of Pathology, H. Lee Moffitt Cancer Center & Research Institute, Tampa, Florida, USA

[11]Department of Biochemistry and Molecular Biology, University of Maryland School of Medicine, Baltimore, Maryland, USA

[*]Corresponding author

Corresponding author email: Eric.Lau@moffitt.org

Supplementary Figure 1

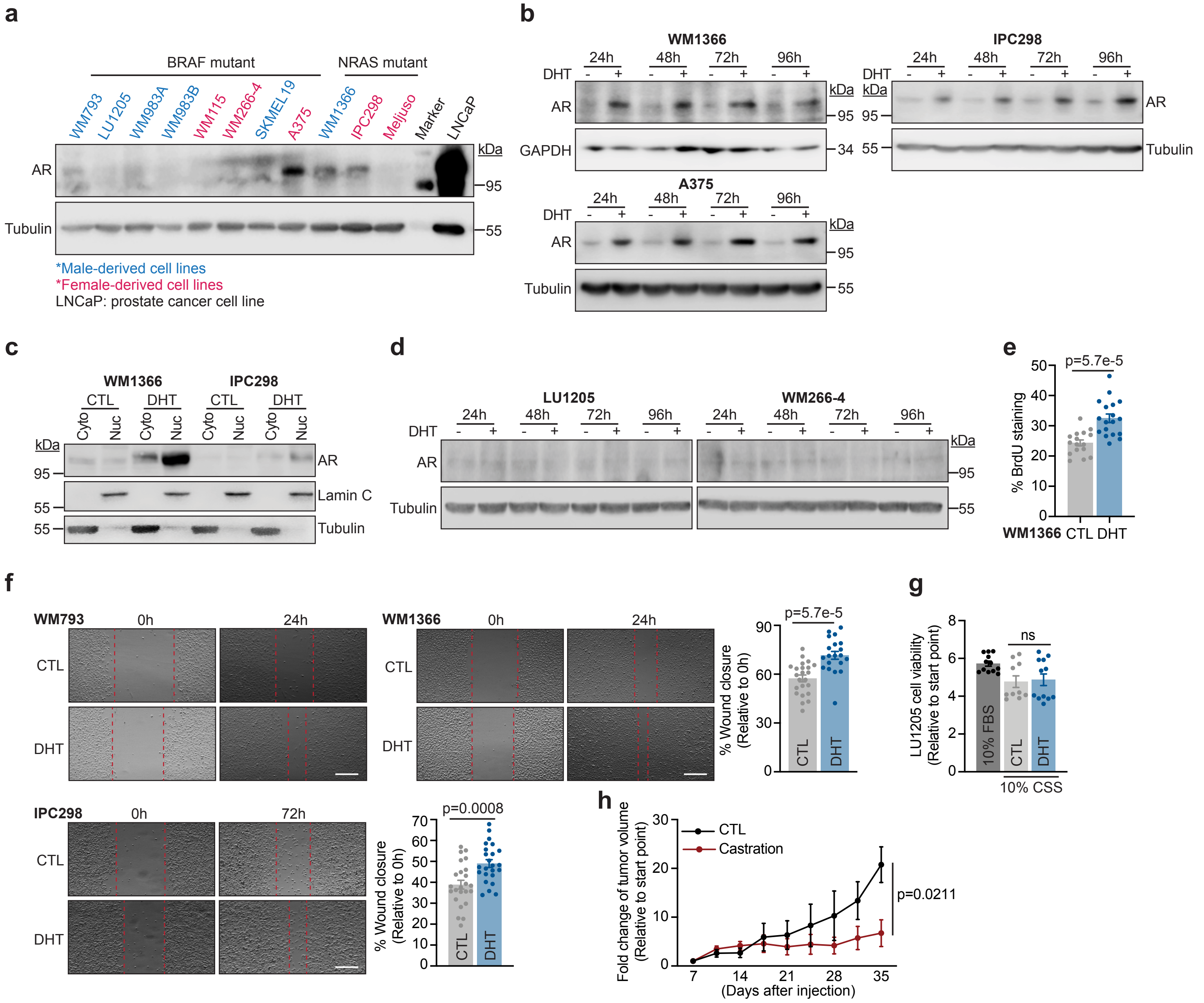

Supplementary Fig. 1: AR$^+$ melanoma cells are responsive to androgen stimulation. (a) Immunoblotting (IB) analysis of baseline AR protein levels across 11 human melanoma cell lines. LNCaP prostate cancer cell line serves as a positive control for AR expression. Uncropped blots in Source Data. **(b)** IB showing the induction of AR protein in WM1366 (*upper left*), IPC298 (*upper right*), and A375 (*lower left*) cells treated ± 100nM DHT over 96hrs. Uncropped blots in Source Data. **(c)** Subcellular fractionation IB of AR protein in WM1366 and IPC298 cells treated ± 100nM DHT for 48hrs. Lamin C and tubulin indicate nuclear (Nuc) and cytoplasmic (Cyto) fractions, respectively. Uncropped blots in Source Data. **(d)** IB showing lack of AR expression and induction in AR$^-$ melanoma cells (LU1205 and WM266-4) treated ± 100nM DHT over 96hrs. Uncropped blots in Source Data. **(e)** BrdU staining of WM1366 cells treated ± 100nM DHT for 48hrs (CTL, n=16 fields; DHT, n=18 fields examined over 3 independent experiments). **(f)** Representative brightfield images for scratch migration assay of WM793 cells (*upper left*; corresponding to **Fig. 1g**, *right*). Representative brightfield images and quantification for WM1366 (*upper right*; CTL, n=23 scratches; DHT, n=20 scratches examined over 2 independent experiments) and IPC298 (*lower left*; n=24 scratches examined over 3 independent experiments) cells treated ± 100nM DHT for 48hrs. Scale bar=400μm. **(g)** MTT assay of LU1205 cells cultured in 10% FBS or 10% CSS ± 100nM DHT for 4 days (10% FBS, n=12; CTL, n=10; DHT, n=12 biologically independently samples). ns, not significant. **(h)** The growth curve of SM1 tumors subcutaneously implanted in CTL or castrated C57BL/6 male mice. Mice were castrated at 1.5 weeks prior to injection (CTL, n=3 mice; Castration, n=5 mice). For **(e)-(h)**, data are presented as mean values ± standard error of the mean (SEM) and *p*-values are calculated by two-sided Student's t-test. Source data are provided as a Source Data file.

Supplementary Figure 2

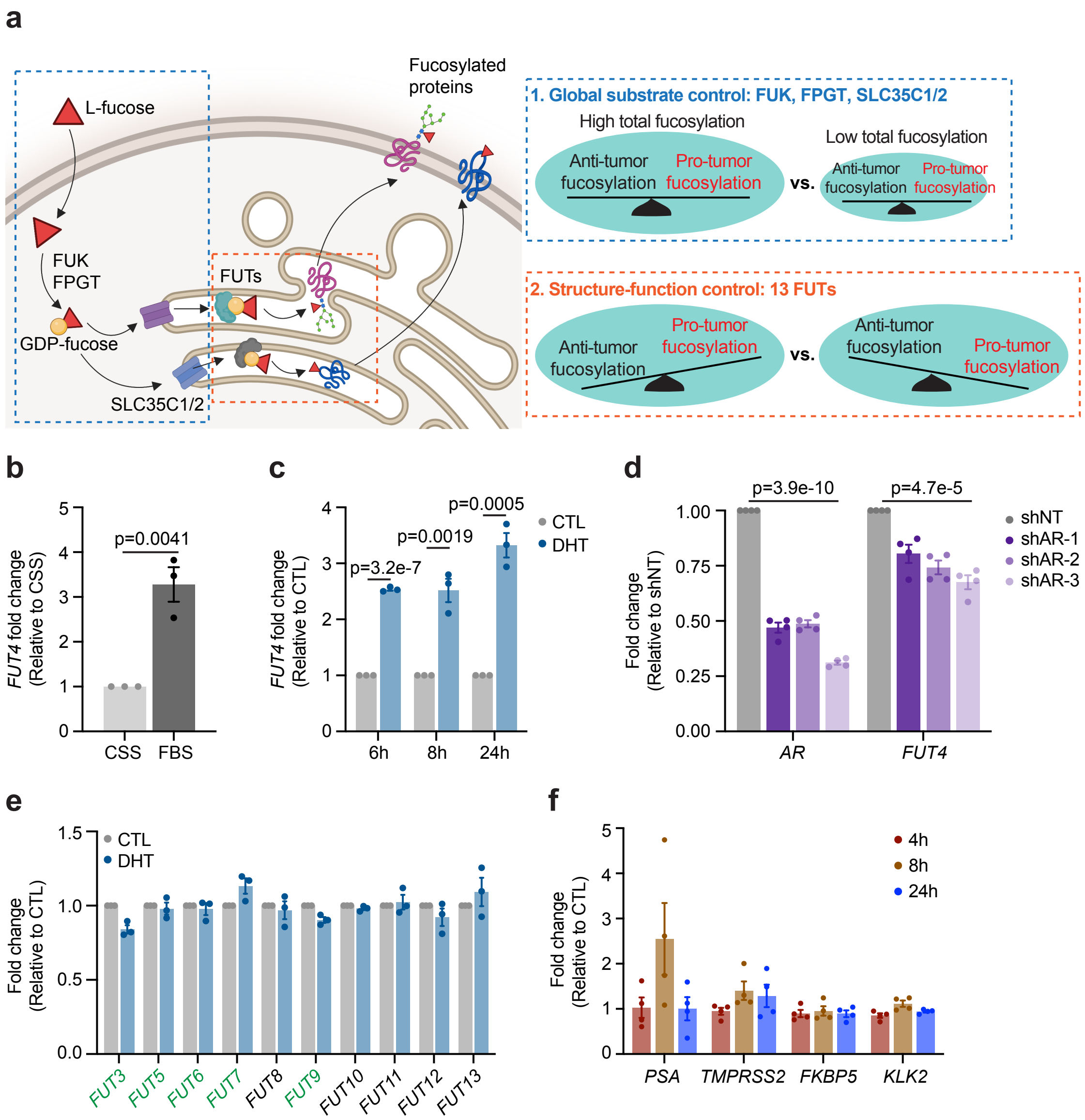

Supplementary Fig. 2: AR transcriptionally upregulates *FUT4*, mediating tumorigenic fucosylation in melanomas. (a) Schematic diagram of fucose salvage pathway: GDP-fucose, the global substrate for cellular protein fucosylation, is transported into ER/Golgi via SLC35C1/2 transporters, where fucose moieties are conjugated onto target proteins by 13 FUTs. FUK, FPGT, and SLC35C1/2 regulate the availability of global substrate (GDP-fucose), whereas the FUTs determine the tumor-promoting vs. tumor-suppressive subtypes of fucosylation (*the schematic was created using BioRender*). qRT-PCR of *FUT4* levels in **(b)** WM793 cells cultured in 10% CSS or 10% FBS for 48hrs (n=3 independent experiments), or in **(c)**, A375 cells treated ± 100nM DHT for 6, 8, and 24hrs (n=3 independent experiments). **(d)** qRT-PCR analysis of *AR* and *FUT4* levels in shNT/shAR A375 cells (n=4 independent experiments). **(e)** qRT-PCR profiling of other *FUTs* in WM793 cells treated ± 100nM DHT for 8hrs (n=3 independent experiments). α-(1,3)-FUTs are indicated with green font. **(f)** qRT-PCR assessment of classical prostate cancer AR targets (*PSA*, *TMPRSS2*, *FKBP5*, and *KLK2*) in WM793 cells treated ± 100nM DHT for 4, 8, and 24hrs (n=4 independent experiments). For **(b)**-**(f)**, data are presented as mean values ± SEM and *p*-values are calculated by two-sided Student's t-test. Source data are provided as a Source Data file.

Supplementary Figure 3

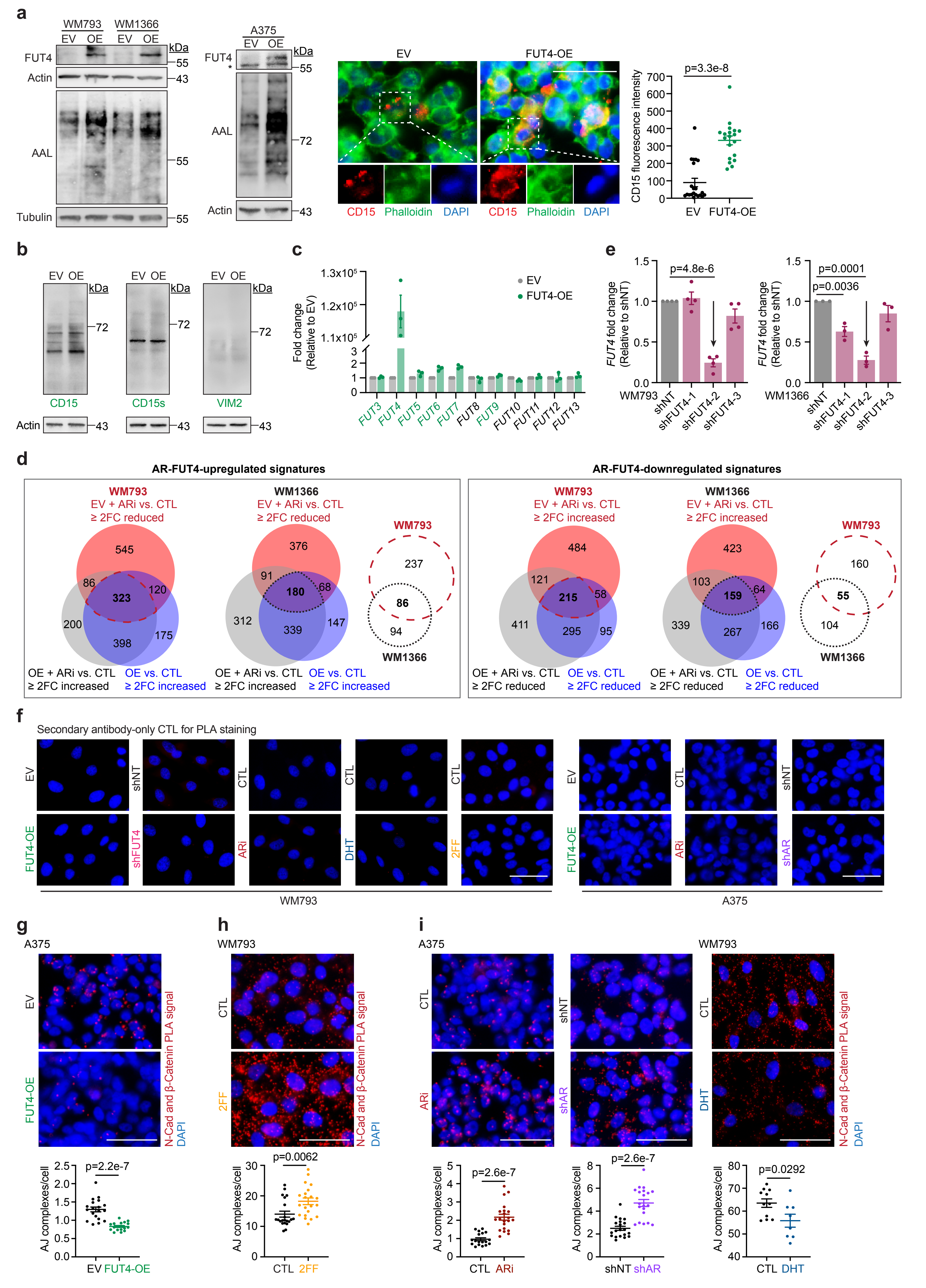

Supplementary Fig. 3: AR-FUT4 axis alters cellular adhesion signaling in melanoma cells. (a) (*left*) IB validation of FUT4 overexpression in WM793, WM1366, and A375 cells. *: non-specific band. Uncropped blots in Source Data. (*right*) IF staining of FUT4-fucosylated epitope (CD15) in EV/FUT4-OE A375 cells (EV, n=21 fields; FUT4-OE, n=19 fields examined over 3 independent experiments). Scale bar=50µm. **(b)** IB analysis of CD15, CD15s, and VIM2 in EV/FUT4-OE A375 cells. Uncropped blots in Source Data. **(c)** qRT-PCR of other *FUTs* in EV/FUT4-OE WM793 cells (n=3 independent experiments). The data confirms the overexpression of *FUT4* in the indicated cell line and shows that *FUT4* overexpression does not impact the expression of other *FUTs*. α-(1,3)-FUTs are indicated with green font. **(d)** Inclusion criteria of AR-FUT4-up/downregulated protein hits overlapped between WM793 and WM1366 cells for Ingenuity Pathway Analysis (IPA). **(e)** qRT-PCR validation of *FUT4* knockdown in WM793 (n=4 independent experiments) and WM1366 (n=3 independent experiments) cells. Arrows indicate shRNAs that were selected based on knockdown efficiency for functional experiments in the remainder of the study. **(f)** Secondary antibody-only control for PLA staining. Scale bar=50µm. PLA staining evaluating the interaction between N-cadherin and β-catenin proteins in **(g)** EV/FUT4-OE A375 cells (EV, n=19 fields; FUT4-OE, n=18 fields examined over 3 independent experiments), **(h)** parental WM793 cells treated ± 250µM 2FF for 3 days (n=21 fields examined over 3 independent experiments), **(i)** (*left*) parental A375 cells treated ± 10µM ARi for 48hrs (CTL, n=18 fields; ARi, n=21 fields examined over 3 independent experiments), (*middle*) shNT/shAR A375 cells (n=20 fields examined over 3 independent experiments), and (*right*) parental WM793 cells treated ± 100nM DHT for 48hrs (CTL, n=11 fields; DHT, n=8 fields examined over 1 independent experiment. Two other independent experiments in Source Data). For **(g)-(i)**, scale bar=50µm. For **(a)**, **(c)**, **(e)**, **(g)-(i)**, data are presented as mean values ± SEM and *p*-values are calculated by two-sided Student's t-test. Source data are provided as a Source Data file.

Supplementary Figure 4

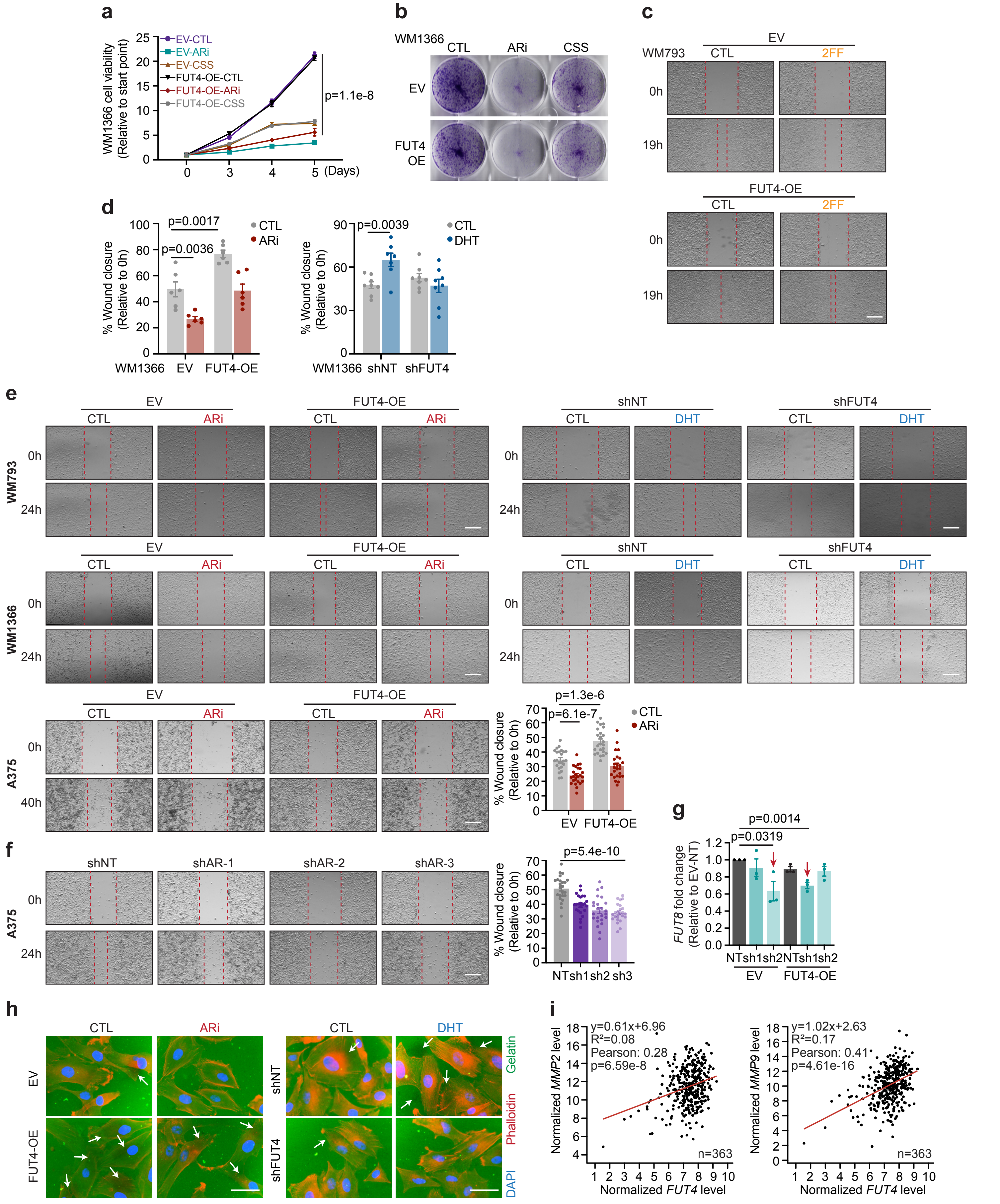

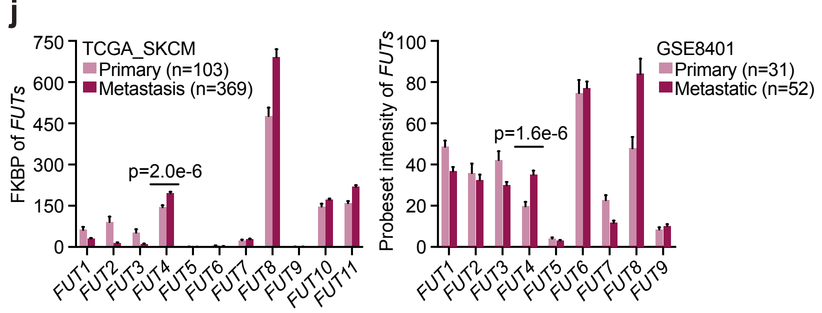

k

Genes	TCGA_SKCM (Log$_2$)			GSE8401 (Log$_2$)		
	p	Bonferroni p adj.	FDR p adj.	p	Bonferroni p adj.	FDR p adj.
FUT4	4.6e-8	0.0009	1.4e-6	1.0e-6	0.0223	1.9e-5
FUT8	4.6e-7	0.009	9.8e-6	0.0002	ns	0.0012
FUT11	4.0e-10	8.1e-6	2.3e-8	-	-	-

Supplementary Fig. 4: FUT4 is crucial for androgen/AR-stimulated melanoma motility *in vitro*. (a) XTT assay (5 days) (n=6 biologically independent samples) and **(b)** clonogenic assay (14 days) of EV/FUT4-OE WM1366 cells treated ± 10μM ARi or ± culture in 10% CSS (representative images are shown for n=3 independent experiments). **(c)** Representative brightfield images of scratch migration assay for EV/FUT4-OE WM793 cells treated ± 250μM 2FF for 3 days in **Fig. 4c**. Scale bar=400μm. **(d)** Scratch migration assay of EV/FUT4-OE WM1366 cells treated ± 10μM ARi (48hrs; *left*; n=6 scratches examined over 1 independent experiment. Another independent experiment in Source Data) or shNT/sh-FUT4 WM1366 cells treated ± 100nM DHT (48hrs; *right*; shNT-CTL, n=8 scratches; shNT-DHT, n=7 scratches; shFUT4-CTL/DHT, n=8 scratches examined over 1 independent experiment. Two other independent experiments in Source Data). **(e)** Representative brightfield images of scratch migration assays for WM793 (*upper*) and WM1366 (*middle*) cells modified/treated as in **Fig. 4d** and **Supplementary Fig. 4d**. (*lower*) Representative brightfield images and quantification of scratch migration assay for EV/FUT4-OE A375 cells treated ± 10μM ARi for 48hrs (n=24 scratches examined over 3 independent experiments). Scale bar=400μm. **(f)** Representative brightfield images and quantification of scratch migration assays for shNT/shAR A375 cells (n=24 scratches examined over 3 independent experiments). Scale bar=400μm. **(g)** qRT-PCR validation of *FUT8* knockdown in EV/FUT4-OE WM793 cells (n=3 independent experiments). **(h)** Representative images of FITC-gelatin degradation assay in **Fig. 4g**. Scale bar=50μm. **(i)** The correlation analysis of *FUT4* and *MMP2/9* levels in TCGA_SKCM cohort. *p*-values are determined by two-sided correlation test based on Pearson's coefficient. **(j)** The comparison of FKBP and Probeset intensity of *FUTs* between primary and metastatic melanomas in TCGA_SKCM and GSE8401 cohorts. **(k)** Bonferroni and FDR tests were applied to the data in **(j)** for the adjustment of *p*-values for multiple hypothesis correction. For **(a)**, **(d)-(g)**, **(j)**, data are presented as mean values ± SEM and *p*-values are calculated by two-sided Student's t-test. Source data are provided as a Source Data file.

Supplementary Figure 5

a

NetNGlyc 1.0: predicted N-glycosylation sites in NP-000416.1

SeqName	Position		Potential	Jury agreement	N-Glyc result
NP_000416.1	100	NNSN	0.7037	(8/9)	+
NP_000416.1	247	NSSS	0.6969	(9/9)	++
NP_000416.1	294	NKTL	0.8157	(9/9)	+++
NP_000416.1	433	NQTY	0.6222	(8/9)	+
NP_000416.1	479	NGTL	0.6889	(9/9)	++
NP_000416.1	490	NDTG	0.5781	(7/9)	+
NP_000416.1	505	NVTI	0.6451	(8/9)	+
NP_000416.1	588	NYSC	0.5812	(7/9)	+
NP_000416.1	671	NQTS	0.6842	(9/9)	++
NP_000416.1	726	NETT	0.6656	(9/9)	++
NP_000416.1	849	NVTY	0.7206	(9/9)	++
NP_000416.1	876	NTTS	0.5368	(7/9)	+
NP_000416.1	979	NLTD	0.6669	(9/9)	++
NP_000416.1	1030	NYSV	0.6076	(8/9)	+
NP_000416.1	1071	NQSS	0.5750	(7/9)	+
NP_000416.1	1105	NGTG	0.5952	(6/9)	+

NetOGlyc 4.0.0.13: predicted O-glycosylation sites in NP-000416.1

SeqName	Position	Score	Comment
NP_000416.1	280	0.512998	#POSITIVE
NP_000416.1	634	0.506116	#POSITIVE
NP_000416.1	706	0.519928	#POSITIVE
NP_000416.1	709	0.5	#POSITIVE
NP_000416.1	1104	0.5	#POSITIVE
NP_000416.1	1107	0.586092	#POSITIVE

b

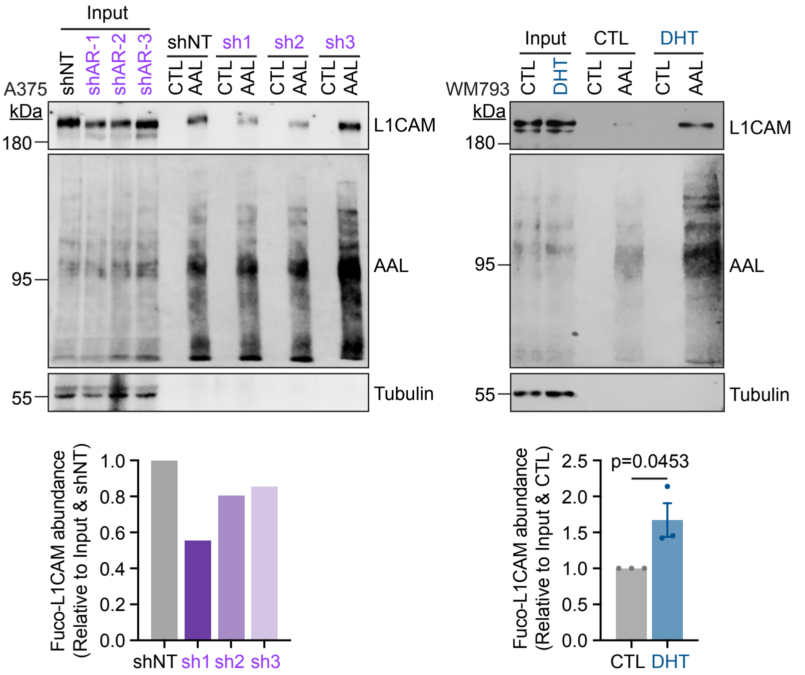

c

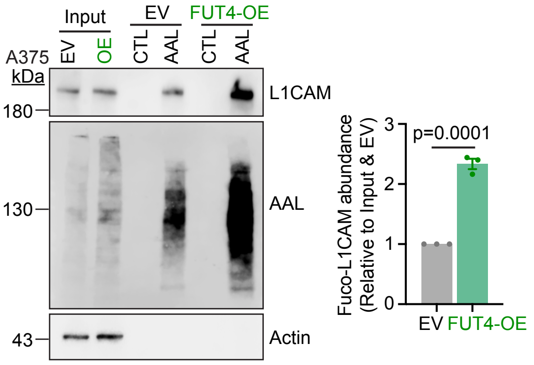

d

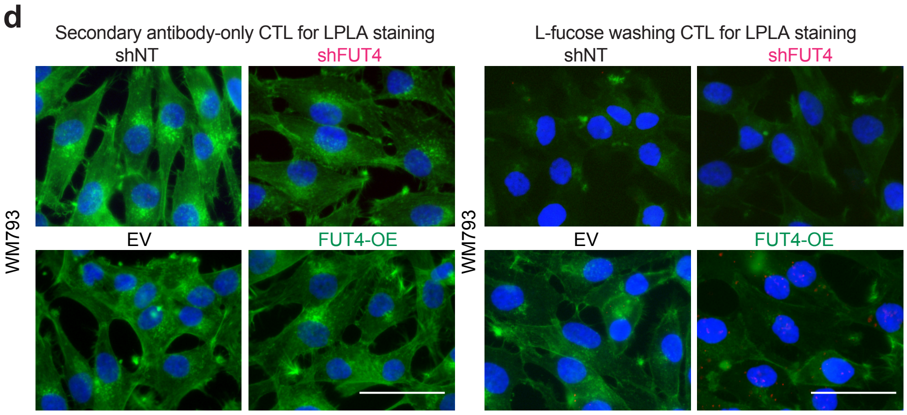

e

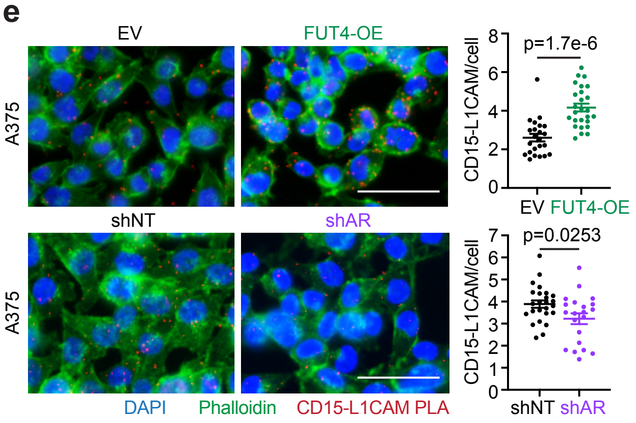

f

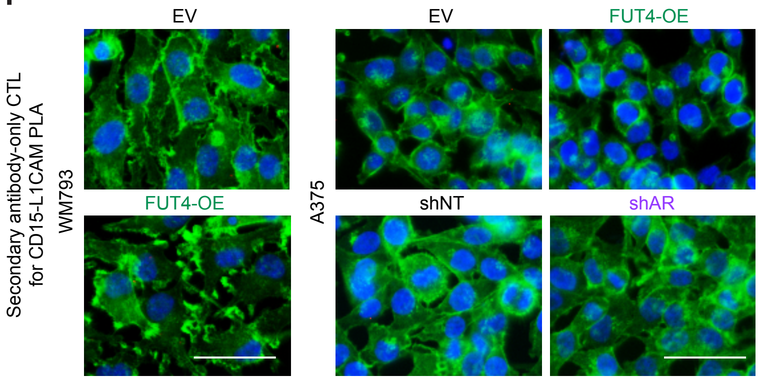

g

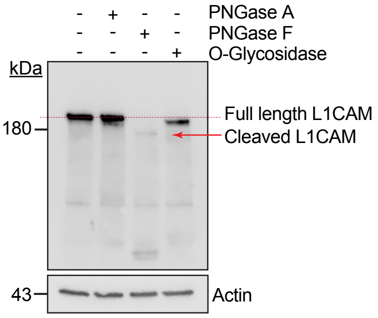

h

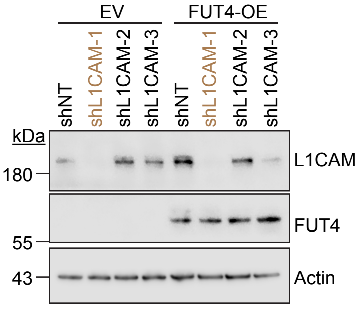

i

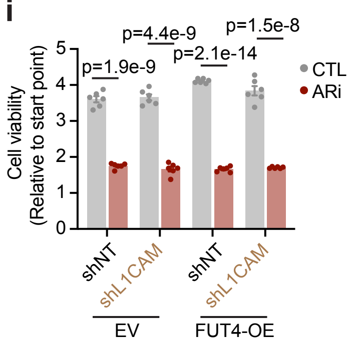

Supplementary Fig. 5: L1CAM is a key downstream effector of AR-FUT4 axis. (a) NetNGlyc and NetOGlyc software predictions of N- and O-glycosylation sites in the L1CAM protein. **(b)** (*upper*) Lectin pulldown (LPD) followed by IB analysis of L1CAM protein in (*left*) shNT/shAR A375 cells and (*right*) parental WM793 cells treated ± 100nM DHT for 48hrs. Column chart (*lower*) shows densitometric quantification for the blots (n=3 independent experiments). Uncropped blots in Source Data. **(c)** LPD followed by IB analysis of L1CAM protein in EV/FUT4-OE A375 cells (n=3 independent experiments). Uncropped blots in Source Data. **(d)** Secondary antibody-only control (*left*) and L-fucose washing control (*right*) for LPLA staining in **Fig. 5c**. Scale bar=50µm. **(e)** PLA staining for CD15 and L1CAM protein in (*upper*) EV/FUT4-OE A375 cells (EV, n=23 fields; FUT4-OE, n=26 fields examined over 3 independent experiments) and (*lower*) shNT/shAR A375 cells (shNT, n=25 fields; shAR, n=21 fields examined over 3 independent experiments). Scale bar=50µm. **(f)** Secondary antibody-only control for CD15-L1CAM PLA staining in **Fig. 5d** and **Supplementary Fig. 5e**. Scale bar=50µm. **(g)** Equal amounts of WM793 lysates subjected to deglycosylation with PNGase A/F and O-Glycosidase enzymes. Reduced L1CAM IB signal for PNGase F and O-glycosidases reflect not reduced L1CAM protein levels but rather reduced recognition by the L1CAM antibody. Uncropped blots in Source Data. **(h)** IB validation of FUT4/L1CAM double-modified WM793 cell lines. Uncropped blots in Source Data. **(i)** XTT assay of FUT4/L1CAM double-modified WM793 cells treated ± 10µM ARi for 6 days (n=6 biologically independent samples). For **(b)**, **(c)**, **(e)**, **(i)**, data are presented as mean values ± SEM and *p*-values are calculated by two-sided Student's t-test. Source data are provided as a Source Data file.

Supplementary Figure 6

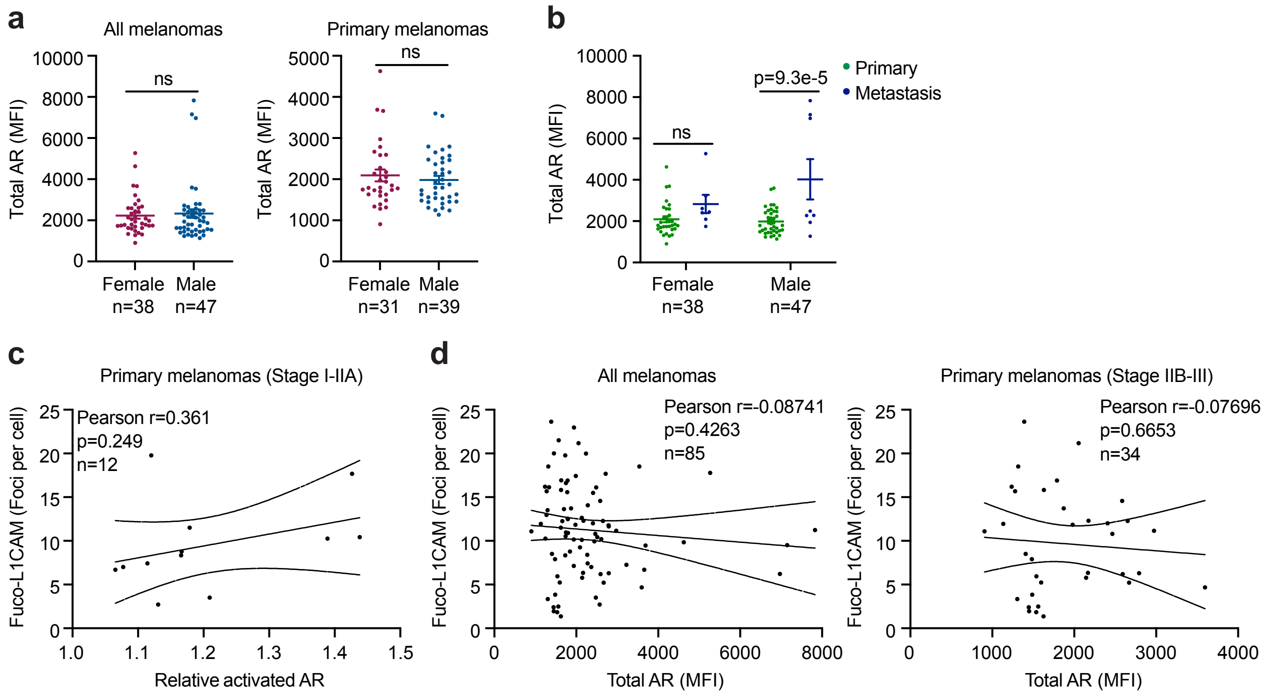

Supplementary Fig. 6: The activation of AR-FUT4-axis in male melanoma tissues. Total AR protein levels in **(a)** female vs. male melanomas or **(b)** primary vs. metastatic melanomas in the melanoma tumor microarray. The correlation analyses of **(c)** activated AR and fuco-L1CAM (LPLA foci) in early-stage (Stage I-IIA) primary melanomas, of **(d)** total AR and fuco-L1CAM (LPLA foci) in all stages (*left*) and late-stage (Stage IIB-III; *right*) melanomas. *p*-values are determined by two-sided correlation test based on Pearson's coefficient. For **(a)** and **(b)**, data are presented as mean values ± SEM and *p*-values are calculated by two-sided Student's t-test. Source data are provided as a Source Data file.

Supplementary Figure 7

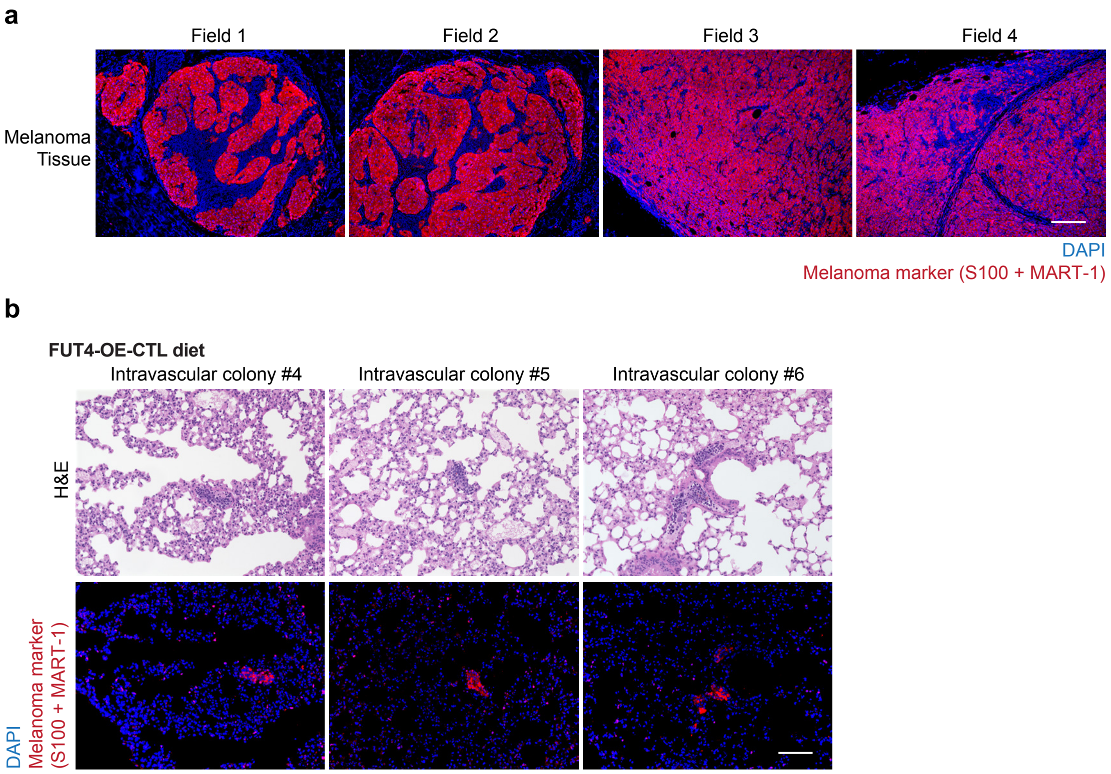

Supplementary Fig. 7: The AR-FUT4 axis promotes accumulation of lung intravascular melanoma colonies *in vivo*. (a) Positive control staining of human melanoma tissues with melanoma marker cocktail (S100 + MART-1). Scale bar=200μm. **(b)** Representative H&E staining and corresponding IF staining of lung intravascular melanoma colonies in mice harboring FUT4-OE melanoma tumors fed with CTL diet. Scale bar=100μm. Source data are provided as a Source Data file.
