# Supplementary material for: Androgen drives melanoma invasiveness and metastatic spread by inducing tumorigenic fucosylation
Source: Nat Commun. 2024 Feb 7;15:1148. doi: 10.1038/s41467-024-45324-w (PMC10850104; doi:10.1038/s41467-024-45324-w)
Supplement: Supplementary file 3 — Description of Additional Supplementary Files [file 41467_2024_45324_MOESM3_ESM.pdf]

Description of Additional Supplementary Files

Supplementary Data 1:

Description: List of primers, key reagents/chemicals, commercial kits, and plasmids used in the study.

Supplementary Data 2:

Description: Matrixes of phosphoproteomics and fucoproteomics data after MaxQuant analysis and IRON or BBSR normalization.
