# Supplementary material for: Androgen drives melanoma invasiveness and metastatic spread by inducing tumorigenic fucosylation
Source: Nat Commun. 2024 Feb 7;15:1148. doi: 10.1038/s41467-024-45324-w (PMC10850104; doi:10.1038/s41467-024-45324-w)
Supplement: Supplementary file 6 — Reporting Summary [file 41467_2024_45324_MOESM6_ESM.pdf]

nature portfolio

Corresponding author(s): Eric K. Lau

Last updated by author(s): Dec 3, 2023

Reporting Summary

Nature Portfolio wishes to improve the reproducibility of the work that we publish. This form provides structure for consistency and transparency in reporting. For further information on Nature Portfolio policies, see our Editorial Policies and the Editorial Policy Checklist.

Statistics

For all statistical analyses, confirm that the following items are present in the figure legend, table legend, main text, or Methods section.

n/a	Confirmed	
☐	☒	The exact sample size (*n*) for each experimental group/condition, given as a discrete number and unit of measurement
☐	☒	A statement on whether measurements were taken from distinct samples or whether the same sample was measured repeatedly
☐	☒	The statistical test(s) used AND whether they are one- or two-sided<br>*Only common tests should be described solely by name; describe more complex techniques in the Methods section.*
☒	☐	A description of all covariates tested
☐	☒	A description of any assumptions or corrections, such as tests of normality and adjustment for multiple comparisons
☐	☒	A full description of the statistical parameters including central tendency (e.g. means) or other basic estimates (e.g. regression coefficient) AND variation (e.g. standard deviation) or associated estimates of uncertainty (e.g. confidence intervals)
☐	☒	For null hypothesis testing, the test statistic (e.g. *F*, *t*, *r*) with confidence intervals, effect sizes, degrees of freedom and *P* value noted<br>*Give P values as exact values whenever suitable.*
☒	☐	For Bayesian analysis, information on the choice of priors and Markov chain Monte Carlo settings
☒	☐	For hierarchical and complex designs, identification of the appropriate level for tests and full reporting of outcomes
☐	☒	Estimates of effect sizes (e.g. Cohen's *d*, Pearson's *r*), indicating how they were calculated

Our web collection on statistics for biologists contains articles on many of the points above.

Software and code

Policy information about availability of computer code

Data collection

For proteomic profiling, a nanoflow ultra-high-performance liquid chromatography (RSLC, Dionex) connected to a Q-Exactive Plus 1035 mass spectrometer (ThermoFisher Scientific) was used for tandem mass spectrometry peptide sequencing, and the data were collected and analyzed using MaxQuant software (version 1.5.2.8.).
Immunoblots were imaged by Image Studio Software on the Odyssey Fc Imaging System ((LI-COR Biosciences).
qRT-PCR was performed using the Bio-Rad CFX Manager 3.1 software on the CFX96 Real-Time PCR Detection System (Bio-Rad).
Absorbances were measured using an iMark microplate absorbance reader (Bio-Rad).
Luciferase activity was measured using GloMax Luminometer Software (Promega).
Immunofluorescently stained TMA slides were imaged with a Zeiss Imager Z2 microscope and Zen software (version 2.3).
The maximum projection TMA images were imported into Definiens Tissue Studio (version 4.7) for TMA core segmentation and quantification.
Brightfield and fluorescence images were captured using the Keyence BZ-X710 microscope or the Leica DMi1 inverted microscope.
TCGA_SKCM data were downloaded from UCSC Xena Functional Genomics Explorer and Broad GDAC Firehose.
GSE8401 microarray dataset was downloaded from NCBI-Gene Expression Omnibus (GEO).

Data analysis

Usage of these software in specific experiments are explained further in the manuscript.
For general statistical analyses, Prism 9 (GraphPad) was used.
For image analyses, Fiji software (NIH) was used.
Gene set enrichment analyses were performed using GSEA software (version 4.2.3).
Pathway enrichment analyses were performed using DAVID (Functional Annotation Tool) and Ingenuity pathway analysis (QIAGEN) platforms.
AR binding scores were predicted with JASPAR database.
Interactome mapping was performed with GeneMANIA.
N-/O-glycosylation sites were predicted on NetNGlyc 1.0 and NetOGlyc 4.0.0.13 servers.

In-house script based on dplyr and tidyverse R packages were used in data frame analysis.
Venn diagrams were generated through BioVenn web application.
TCGA_SKCM co-expression analysis was performed with cBioPortal.

For manuscripts utilizing custom algorithms or software that are central to the research but not yet described in published literature, software must be made available to editors and reviewers. We strongly encourage code deposition in a community repository (e.g. GitHub). See the Nature Portfolio guidelines for submitting code & software for further information.

Data

Policy information about availability of data

All manuscripts must include a data availability statement. This statement should provide the following information, where applicable:

- Accession codes, unique identifiers, or web links for publicly available datasets
- A description of any restrictions on data availability
- For clinical datasets or third party data, please ensure that the statement adheres to our policy

The mass spectrometry proteomics data have been deposited to the ProteomeXchange Consortium via the PRIDE partner repository with the dataset identifiers PXD047335 (Phosphoproteomics) and PXD047337 (Fucoproteomics). Initial matrixes of proteomics data after MaxQuant analysis and IRON or BBSR normalization are provided in Supplementary Data 2. The main data supporting the findings of this study are available within the article and its Supplementary Information files. In-house script based on dplyr and tidyverse R packages (https://github.com/tidyverse/dplyr) were used in data frame analysis. TCGA dataset was downloaded from UCSC Xena Functional Genomics Explorer (https://xenabrowser.net) and Broad GDAC Firehose (https://gdac.broadinstitute.org). GSE8401 microarray dataset was downloaded from NCBI-Gene Expression Omnibus (GEO, https://www.ncbi.nlm.nih.gov/geo/query/acc.cgi?acc=GSE8401). All antibodies/software used in this study are listed in Reporting Summary. All primer sequences/special chemicals and reagents/commercial kits/plasmids applied in this study are listed in Supplementary Data 1. Source data are provided with this paper. Additional data are available from the corresponding author upon reasonable request.

Research involving human participants, their data, or biological material

Policy information about studies with human participants or human data. See also policy information about sex, gender (identity/presentation), and sexual orientation and race, ethnicity and racism.

Reporting on sex and gender	This study focuses on delineating the cellular and molecular mechanisms underlying sex-associated disparities in melanoma pathogenesis, where male patients historically exhibit higher incidence and worse outcomes as compared to female patients. Our study specifically aimed to elucidate how the male sex hormone (androgen) drives melanoma malignancy contributing to poor prognosis in male patients. Therefore, sex is a prominent factor in this study. The tumor microarray used is a commercial product from US Biomax (#ME1004h) and is subject to commercial inclusion criteria beyond our control. However, it includes specimens from 42 female and 58 male patients, which has the approximately equal representation of female and male sex. In addition, the human melanoma cell lines used in this study represent both male and female patient donors as indicated in the text.
Reporting on race, ethnicity, or other socially relevant groupings	NA
Population characteristics	The tumor microarray used in Fig. 6 and Supplementary Fig. 6 is a commercial product from US Biomax (#ME1004h). This microarray includes specimens from 42 female and 58 male; age range: 21 day-84; specimen types: 2 normal skins, 14 benign skins, 62 malignant melanomas (Stage IA-III), 22 metastatic melanomas. The human melanoma cell lines used in this study represent both male and female patients as indicated in the text.
Recruitment	The tumor microarray used in Fig. 6 and Supplementary Fig. 6 is a commercial product from US Biomax (#ME1004h). Therefore, the construction of the microarray was subject to availability and out of our control. We chose this particular microarray as it included both primary and metastatic melanoma specimens with relatively even representation of female vs. male patients, staging, and tumor site.
Ethics oversight	The commercial human melanoma tissue microarrays were purchased from US Biomax (#ME1004h), with an ethics statement, "All tissue is collected under the highest ethical standards with the donor being informed completely and with their consent. We make sure we follow standard medical care and protect the donors' privacy. All human tissues are collected under HIPPA approved protocols. All animal tissues are collected under IACUC protocol. All samples have been tested negative for HIV and Hepatitis B or their counterparts in animals and approved for commercial product development".

Note that full information on the approval of the study protocol must also be provided in the manuscript.

Field-specific reporting

Please select the one below that is the best fit for your research. If you are not sure, read the appropriate sections before making your selection.

☒ Life sciences ☐ Behavioural & social sciences ☐ Ecological, evolutionary & environmental sciences

For a reference copy of the document with all sections, see nature.com/documents/nr-reporting-summary-flat.pdf

Life sciences study design

All studies must disclose on these points even when the disclosure is negative.

Sample size	For in vivo experiment, we planned to implement 10 mice per group. With this number, we estimated the ability to detect a 10% difference in tumor development between any 2 conditions with a p-value of 0.05 and a power of 0.80, and a 20% change with a p-value of 0.05 and a power of 0.95. In our experience, 10 mice per group have been more than sufficient to provide statistical power and buffer for incidental loss of mice in the cohort (e.g., unexpected death, tumor ulceration/did not graft successfully).<br>For in vitro experiments, the number of samples/conditions analyzed are described in the Figure Legends. Sample size was not predetermined statistically but was chosen based on previous experience and published literatures to ensure adequate statistical power and obtain statistically relevant results.
Data exclusions	For in vivo experiment, mice died incidentally in the middle of the experiment or mice with failed tumor engraftment were excluded from the cohort. In TMA analysis, certain cores with poor or no DAPI staining were excluded from the analysis.
Replication	All western blots were performed at least twice; findings from given western blots were consistent with western blots derived from other cell types, etc. (thus providing further replication of data between figure panels). All in vitro experiments are representative of at least three independent replicates with consistent and reproducible results. All attempts were successful. The detailed replicate information was provided in the Figure Legends and Source Data file. For proteomic profiling, mass spectrometry was performed once or twice on samples pooled from 3 biological replicates. Subsequent validation was performed in biological triplicate samples.
Randomization	For all experiments, samples/cells/mice were randomly allocated into control and treatment groups.
Blinding	Proteomics data were collected blindly by the Proteomics and Metabolomics Core at Moffitt Cancer Center & Research Institute. No blinding was performed for the other experiments considering the complexity of the study as well as the unbiased data collection/analysis/statistical tests performed with certified software.

Reporting for specific materials, systems and methods

We require information from authors about some types of materials, experimental systems and methods used in many studies. Here, indicate whether each material, system or method listed is relevant to your study. If you are not sure if a list item applies to your research, read the appropriate section before selecting a response.

Materials & experimental systems

n/a	Involved in the study	
☐	☒	Antibodies
☐	☒	Eukaryotic cell lines
☒	☐	Palaeontology and archaeology
☐	☒	Animals and other organisms
☒	☐	Clinical data
☒	☐	Dual use research of concern
☒	☐	Plants

Methods

n/a	Involved in the study	
☒	☐	ChIP-seq
☒	☐	Flow cytometry
☒	☐	MRI-based neuroimaging

Antibodies

Antibodies used

Rabbit monoclonal anti-AR
•Cell Signaling Technology; Cat# 5153S; Clone# D6F11; Lot# 9
•Applications: WB (1:1000; 82ng/ml), IF (1:200; 410ng/ml), ChIP (1.5µg/IP reaction), IF-Paraffin (1:50; 1.64µg/ml).

Mouse monoclonal anti-N-Cadherin
•Novus Biologicals; Cat# NBP1-48309; Clone# 13A9; Lot# C-3
•Applications: WB (1:1000; 1µg/ml), IP (1.5µg/reaction), PLA (1:125; 8µg/ml), PLA-Paraffin (1:50; 20µg/ml)

Rabbit monoclonal anti-β-Catenin
•Cell Signaling Technology; Cat# 8480S; Clone# D10A8; Lot# 9
•Applications: WB (1:1000; 6ng/ml), PLA (1:125; 48ng/ml), PLA-Paraffin (1:100; 60ng/ml)

Rabbit monoclonal anti-Catenin δ-1
•Cell Signaling Technology; Cat# 59854S; Clone# D7S2M; Lot# 1
•Applications: PLA (1:125; 2.12µg/ml)

Mouse monoclonal anti-L1CAM
•Novus Biologicals; Cat# NB100-2682; Clone# UJ127.11; Lot# CMSD02-4
•Applications: WB (1:500; 2µg/ml), LPLA (1:200; 5µg/ml), LPLA-Paraffin (1:100; 10µg/ml), IF-Paraffin (1:100; 10µg/ml)

Rabbit monoclonal anti-NCAM-L1
•Cell Signaling Technology; Cat# 89861S; Clone# D5N9S; Lot# 1

•Applications: WB (1:500; 178ng/ml)

Mouse monoclonal anti-FUT4
•Abcam; Cat# ab181461; Clone# 6B11B4; Lot# GR170435-23
•Applications: WB (1:500; 2μg/ml)

Rabbit monoclonal anti-CD15
•Novus Biologicals; Cat# NBP3-12070; Clone# BRA-4F1; Lot# T2041D15
•Applications: IF (1:200; 5μg/ml), PLA (1:150; 6.7μg/ml), WB (1:500; 2μg/ml)

Mouse monoclonal anti-CD15s
•BD Biosciences; Cat# 551344; Clone# CSLEX1; Lot# 7311761
•Applications: WB (1:500; 1μg/ml)

Mouse monoclonal anti-CD65s
•Nordic MUbio; Cat# GM4101; Clone# VIM2; Lot# 16568
•Applications: WB (1:500; 0.4μg/ml)

Alexa Fluor 488 Melanoma Marker (MART-1 + Tyrosinase + gp100)
•Novus Biologicals; Cat# NBP2-34681AF488; Clone# M2-7C10 + M2-9E3 + T311 + HMB45; Lot# D111943
•Applications: IF-Paraffin (1:100; 2μg/ml)

Rabbit polyclonal anti-MART-1
•Sigma-Aldrich; Cat# SAB4500949; Clone# NA; Lot# 310257
•Applications: IF-Paraffin (1:200; 5μg/ml)

Rabbit polyclonal anti-S100
•Agilent; Cat# GA50461-2; Clone# NA; Lot# 41387851
•Applications: IF-Paraffin (Ready-to-use; no dilution needed)

Mouse anti-beta tubulin
•Developmental Studies Hybridoma Bank; Cat# E7; Clone# E7; Lot# 11/29/18
•Applications: WB (1:500; 54ng/ml)

Mouse monoclonal anti-actin
•Developmental Studies Hybridoma Bank; Cat# JLA20; Clone# JLA20; Lot# NA
•Applications: WB (1:1000)

Mouse monoclonal anti-GAPDH
•Invitrogen; Cat# MA5-15738; Clone# GA1R; Lot# XD341955
•Applications: WB (1:1000; 1μg/ml)

Goat polyclonal anti-Lamin A/C
•Santa Cruz Biotechnology; Cat# sc-6215; Clone# N-18; Lot# C2113
•Applications: WB (1:500; 0.2μg/ml)

Biotinylated AAL
•Vector Laboratories; Cat# B-1395-1; Clone# NA; Lot# ZJ0827
•Applications: WB (1:1000; 2μg/ml), LPLA (1:200; 10μg/ml), LPLA-Paraffin (1:100; 20μg/ml)

Rabbit monoclonal anti-IgG isotype control
•Cell Signaling Technology; Cat# 3900S; Clone# DA1E; Lot# 50
•Applications: IP (1.5μg/reaction)

Mouse monoclonal anti-BrdU
•Sigma-Aldrich; Cat# B8434; Clone# BU-33; Lot# 0000222427
•Applications: IF (1:500; 3μg/ml)

Alexa Fluor 488 Phalloidin
•Invitrogen; Cat# A12379; Clone# NA; Lot# 1737901
•Applications: IF (1:50; 1unit/coverslip)

Alexa Fluor 594 Phalloidin
•Invitrogen; Cat# A12381; Clone# NA; Lot# 2256805
•Applications: IF (1:50; 1unit/coverslip)

m-IgGκ BP-HRP
•Santa Cruz Biotechnology; Cat# sc-516102; Lot# B2221
•Applications: WB (1:5000; 80ng/ml)

mouse anti-rabbit IgG-HRP
•Santa Cruz Biotechnology; Cat# sc-2357; Lot# G0720
•Applications: WB (1:5000; 80ng/ml)

mouse anti-goat IgG-HRP
•Santa Cruz Biotechnology; Cat# sc-2354; Lot# F0722

•Applications: WB (1:5000; 80ng/ml)

IRDye 680RD Goat anti-Mouse IgG
•LI-COR; Cat# 926-68070; Lot# C50113-05
•Applications: WB (1:5000; 0.2μg/ml)

Streptavidin Protein, DyLight 800
•Invitrogen; Cat# 21851; Lot# TD268565
•Applications: WB (1:5000; 0.2μg/ml)

Goat anti-biotin
•Vector Laboratories; Cat# SP-3000; Lot# ZH0202
•Applications: LPLA (1:400; 2.5μg/ml), LPLA-Paraffin (1:200; 5μg/ml)

Goat anti-Mouse Alexa Fluor 568
•Invitrogen; Cat# A-11004; Lot# 2090670
•Applications: IF (1:250; 8μg/ml)

Donkey anti-Rabbit Alexa Fluor 568
•Invitrogen; Cat# A-10042; Lot# 2207536
•Applications: IF (1:250; 8μg/ml), IF-Paraffin (1:200; 10μg/ml)

Donkey anti-Mouse Alexa Fluor 647
•Invitrogen; Cat# A-31571; Lot# 1476600
•Applications: IF (1:250; 8μg/ml)

Goat anti-Rabbit Alexa Fluor 647
•Invitrogen; Cat# A-32733; Lot# UH283999
•Applications: IF (1:250; 8μg/ml)

Validation

All primary antibodies used are commercially available and were applied per manufacturer instructions. The validation of each primary antibody for the species and application was performed by the provider, as indicated on the manufacturer website. The applications of antibodies were also validated across multiple other publications, with references listed on the manufacturer website.

Rabbit monoclonal anti-AR
•Website: https://www.cellsignal.com/products/primary-antibodies/androgen-receptor-d6f11-xp-rabbit-mab/5153
•Manufacturer verified for use: WB, IP, IHC, IF, Flow, ChIP
•Relevant citations: Salah-Eddine Lamhamedi-Cherradi, et al. The androgen receptor is a therapeutic target in desmoplastic small round cell sarcoma. Nat Commun. 2022 Jun 1;13(1):3057. PMID: 35650195.
Nader Al-Nakouzi, et al. Reformation of the chondroitin sulfate glycocalyx enables progression of AR-independent prostate cancer. Nat Commun. 2022 Aug 13;13(1):4760. PMID: 35963852.

Mouse monoclonal anti-N-Cadherin
•Website: https://www.novusbio.com/products/n-cadherin-antibody-13a9_nbp1-48309
•Manufacturer verified for use: WB, Simple Western, Flow, Flow-IC, ICC/IF, IHC, IHC-P, IP, ICC/IF
•Relevant citations: James K Wahl 3rd, et al. N-cadherin-catenin complexes form prior to cleavage of the proregion and transport to the plasma membrane. J Biol Chem. 2003 May 9;278(19):17269-76. PMID: 12604612.
S Islam, et al. Expression of N-cadherin by human squamous carcinoma cells induces a scattered fibroblastic phenotype with disrupted cell-cell adhesion. J Cell Biol. 1996 Dec;135(6 Pt 1):1643-54. PMID: 8978829.

Rabbit monoclonal anti-β-Catenin
•Website: https://www.cellsignal.com/products/primary-antibodies/b-catenin-d10a8-xp-rabbit-mab/8480
•Manufacturer verified for use: WB, IP, IHC, IF, Flow, ChIP, CUT&RUN
•Relevant citations: Regina Padmanabhan, et al. Quantification of the growth suppression of HER2+ breast cancer colonies under the effect of trastuzumab and PD-1/PD-L1 inhibitor. Front Oncol. 2022 Dec 7;12:977664. PMID: 36568154.
Jiadi Lv, et. al. Cell softness regulates tumorigenicity and stemness of cancer cells. EMBO J. 2021 Jan 15;40(2):e106123. PMID: 33274785.

Rabbit monoclonal anti-Catenin δ-1
•Website: https://www.cellsignal.com/products/primary-antibodies/catenin-d-1-d7s2m-xp-rabbit-mab/59854
•Manufacturer verified for use: WB, IP, IHC, IF
•Relevant citations: Stephanie A Sheehan, et al. Heterocellular N-cadherin junctions enable nontransformed cells to inhibit the growth of adjacent transformed cells. Cell Commun Signal. 2022 Feb 17;20(1):19. PMID: 35177067.
Ki-Sook Park, et al. Enhanced endothelial barrier function by monoclonal antibody activation of vascular endothelial cadherin. Am J Physiol Heart Circ Physiol. 2021 Apr 1;320(4):H1403-H1410. PMID: 33577432.

Mouse monoclonal anti-L1CAM
•Website: https://www.novusbio.com/products/l1cam-antibody-uj12711_nb100-2682
•Manufacturer verified for use: WB, ELISA, Flow, Flow-CS, ICC/IF, IHC, IHC-Fr, IHC-P, IP, CyTOF-ready, ICC/IF
•Relevant citations: Ran Tomomasa, et al. Ependymoma-like tumor with mesenchymal differentiation harboring C11orf95-NCOA1/2 or -RELA fusion: A hitherto unclassified tumor related to ependymoma. Brain Pathol. 2021 May;31(3):e12943. PMID: 33576087.
Atsushi Sasaki, et al. Review of ependymomas: assessment of consensus in pathological diagnosis and correlations with genetic profiles and outcome. Brain Tumor Pathol. 2019 Apr;36(2):92-101. PMID: 30929114.

Rabbit monoclonal anti-NCAM-L1

•Website: https://www.cellsignal.com/products/primary-antibodies/ncam-l1-d5n9s-rabbit-mab/89861
•Manufacturer verified for use: WB, IP, IF
•Relevant citations: Courtney M McKernan, et. al. ABL kinases regulate translation in HER2+ cells through Y-box-binding protein 1 to facilitate colonization of the brain. Cell Rep. 2022 Aug 30;40(9):111268. PMID: 36044842.
Marzia Perluigi, et. al. Aberrant crosstalk between insulin signaling and mTOR in young Down syndrome individuals revealed by neuronal-derived extracellular vesicles. Alzheimers Dement. 2022 Aug;18(8):1498-1510. PMID: 34812584.

Mouse monoclonal anti-FUT4
•Website: https://www.abcam.com/products/primary-antibodies/fut4-antibody-6b11b4-ab181461.html
•Manufacturer verified for use: WB
•Relevant citations: Yintai Li, et al. MicroRNA-200b relieves LPS-induced inflammatory injury by targeting FUT4 in knee articular chondrocytes in vitro. Exp Ther Med. 2021 Apr;21(4):407. PMID: 33692838.
Aman Wang, et al. Tumor-associated macrophages promote Ezrin phosphorylation-mediated epithelial-mesenchymal transition in lung adenocarcinoma through FUT4/LeY up-regulation. Oncotarget. 2017 Apr 25;8(17):28247-28259. PMID: 28423676.

Rabbit monoclonal anti-CD15
•Website: https://www.novusbio.com/products/cd15-lewis-x-antibody-bra-4f1_nbp3-12070
•Manufacturer verified for use: Flow, ICC/IF, IHC, IP
•Relevant citations: NA

Mouse monoclonal anti-CD15s
•Website: https://www.bdbiosciences.com/en-us/products/reagents/flow-cytometry-reagents/research-reagents/single-color-antibodies-ruo/purified-mouse-anti-human-cd15s.551344
•Manufacturer verified for use: Flow, WB, ELISA/RIA, Immunostaining
•Relevant citations: NA

Mouse monoclonal anti-CD65s
•Website: https://nordicmubio.com/products/mouse-anti-cd65s/GM-4101
•Manufacturer verified for use: IF, Flow
•Relevant citations: NA

Alexa Fluor 488 Melanoma Marker (MART-1 + Tyrosinase + gp100)
•Website: https://www.novusbio.com/products/melanoma-marker-mart-1-tyrosinase-gp100-antibody-m2-7c10-m2-9e3-t311-hmb45_nbp2-34681af488
•Manufacturer verified for use: WB, ICC/IF, IHC, IHC-Fr, IHC-P
•Relevant citations: Jan Martinek, et al. Transcriptional profiling of macrophages in situ in metastatic melanoma reveals localization-dependent phenotypes and function. Cell Rep Med. 2022 May 17;3(5):100621. PMID: 35584631.

Rabbit polyclonal anti-MART-1
•Website: https://www.sigmaaldrich.com/US/en/product/sigma/sab4500949
•Manufacturer verified for use: ELISA, IHC, WB
•Relevant citations: D Valmori, et al. Enhanced generation of specific tumor-reactive CTL in vitro by selected Melan-A/MART-1 immunodominant peptide analogues. J Immunol. 1998 Feb 15;160(4):1750-8. PMID: 9469433
Toshihiko Hoashi, et al. MART-1 is required for the function of the melanosomal matrix protein PMEL17/GP100 and the maturation of melanosomes. J Biol Chem. 2005 Apr 8;280(14):14006-16. PMID: 15695812.

Rabbit polyclonal anti-S100
•Website: https://www.agilent.com/en/product/immunohistochemistry/antibodies-controls/primary-antibodies/s100-%28dako-omnis%29-76198
•Manufacturer verified for use: IHC
•Relevant citations: NA

Mouse anti-beta tubulin
•Website: https://dshb.biology.uiowa.edu/E7_2
•Manufacturer verified for use: IHC, IF, ICC, WB
•Relevant citations: Megan I Brasher, et al. Interaction of Munc18c and syntaxin4 facilitates invadopodium formation and extracellular matrix invasion of tumor cells. J Biol Chem. 2017 Sep 29;292(39):16199-16210. PMID: 28798239.
Sung-Tae Hong, et al. Antagonistic roles of Drosophila Tctp and Brahma in chromatin remodelling and stabilizing repeated sequences. Nat Commun. 2016 Sep 30;7:12988. PMID: 27687497.

Mouse monoclonal anti-actin
•Website: https://dshb.biology.uiowa.edu/JLA20
•Manufacturer verified for use: IHC, IF, ICC, WB
•Relevant citations: Jun Wan, et al. Mad1 destabilizes p53 by preventing PML from sequestering MDM2. Nat Commun. 2019 Apr 4;10(1):1540. PMID: 30948704.
Rebekah Mahoney, et al. Pathogenic Tau Causes a Toxic Depletion of Nuclear Calcium. Cell Rep. 2020 Jul 14;32(2):107900. PMID: 32668249.

Mouse monoclonal anti-GAPDH
•Website: https://www.thermofisher.com/antibody/product/GAPDH-Loading-Control-Antibody-clone-GA1R-Monoclonal/MA5-15738
•Manufacturer verified for use: WB, IHC, ICC/IF, Flow, ELISA, IP, FN, PLA, Misc
•Relevant citations: Eunjin Oh, et al. Munc18-1 regulates first-phase insulin release by promoting granule docking to multiple syntaxin isoforms. J Biol Chem. 2012 Jul 27;287(31):25821-33. PMID: 22685295.
Zilai Wang, et al. Cell Lineage-Based Stratification for Glioblastoma. Cancer Cell. 2020 Sep 14;38(3):366-379.e8. PMID: 32649888.

Goat polyclonal anti-Lamin A/C

•Website: https://www.scbt.com/p/lamin-a-c-antibody-n-18
•Manufacturer verified for use: WB, IP, IF, IHC, ELISA
•Relevant citations: K-W Min, et al. NAG-1/GDF15 accumulates in the nucleus and modulates transcriptional regulation of the Smad pathway. Oncogene. 2016 Jan 21;35(3):377-88. PMID: 25893289.
Chin Yee Ho, et al. Lamin A/C and emerin regulate MKL1-SRF activity by modulating actin dynamics. Nature. 2013 May 23;497(7450):507-11. PMID: 23644458.

Biotinylated AAL
•Website: https://vectorlabs.com/products/glycobiology/biotinylated-aleuria-aurantia-lectin-aal
•Manufacturer verified for use: IHC/ICC, IF, WB, Elispot, ELISA, Glycobiology
•Relevant citations: Li Zhu, et al. Structural insights into mechanism and specificity of the plant protein O-fucosyltransferase SPINDLY. Nat Commun. 2022 Dec 2;13(1):7424. PMID: 36456586.
Edyta Skurska, et al. Incorporation of fucose into glycans independent of the GDP-fucose transporter SLC35C1 preferentially utilizes salvaged over de novo GDP-fucose. J Biol Chem. 2022 Aug;298(8):102206. PMID: 35772493.

Rabbit monoclonal anti-IgG isotype control
•Website: https://www.cellsignal.com/products/primary-antibodies/rabbit-da1e-mab-igg-xp-isotype-control/3900
•Manufacturer verified for use: IP, IHC, IF, Flow, ChIP
•Relevant citations: Justine Marsolier, et al. H3K27me3 conditions chemotolerance in triple-negative breast cancer. Nat Genet. 2022 Apr;54(4):459-468. PMID: 35410383.
Erik S Knudsen, et al. CDK/cyclin dependencies define extreme cancer cell-cycle heterogeneity and collateral vulnerabilities. Cell Rep. 2022 Mar 1;38(9):110448. PMID: 35235778.

Mouse monoclonal anti-BrdU
•Website: https://www.sigmaaldrich.com/US/en/product/sigma/b8434
gclid=Cj0KCQiAgaGgBhC8ARIsAAAyLfH_Q3cA2sJ3x72LQiCaKl9wkaeCroWqDV-bn6xAEq39325J5mMU1gQaAtolEALw_wcB&gclsrc=aw.ds
•Manufacturer verified for use: IHC, immunostaining
•Relevant citations: Yan Cheng, et al. NeuroD1 Dictates Tumor Cell Differentiation in Medulloblastoma. Cell Rep. 2020 Jun 23;31(12):107782. PMID: 32579914.
Chenglu Xiao, et al. Chromatin-remodelling factor Brg1 regulates myocardial proliferation and regeneration in zebrafish. Nat Commun. 2016 Dec 8;7:13787. PMID: 27929112.

Alexa Fluor 488 Phalloidin
•Website: https://www.thermofisher.com/order/catalog/product/A12379
•Manufacturer verified for use: IF
•Relevant citations: Thomas H Barker, et al. SPARC regulates extracellular matrix organization through its modulation of integrin-linked kinase activity. J Biol Chem. 2005 Oct 28;280(43):36483-93. PMID: 16115889.
Ama Gassama-Diagne, et al. Phosphatidylinositol-3,4,5-trisphosphate regulates the formation of the basolateral plasma membrane in epithelial cells. Nat Cell Biol. 2006 Sep;8(9):963-70. PMID: 16921364.

Alexa Fluor 594 Phalloidin
•Website: https://www.thermofisher.com/order/catalog/product/A12381
•Manufacturer verified for use: IF
•Relevant citations: Ashish C Massey, et al. Consequences of the selective blockage of chaperone-mediated autophagy. Proc Natl Acad Sci U S A. 2006 Apr 11;103(15):5805-10. PMID: 16585521.
R A Rebres, et al. Membrane raft association of CD47 is necessary for actin polymerization and protein kinase C theta translocation in its synergistic activation of T cells. J Biol Chem. 2001 Mar 9;276(10):7672-80. PMID: 11114301.

Goat anti-biotin
•Website: https://vectorlabs.com/products/antibodies/goat-anti-biotin-unconjugated#biozbadges
•Manufacturer verified for use: IHC/ICC, IF, in situ hybridization, WB, Elispot, ELISA
•Relevant citations: Takeo Isozaki, et al. Fucosyltransferase 1 mediates angiogenesis, cell adhesion and rheumatoid arthritis synovial tissue fibroblast proliferation. Arthritis Res Ther. 2014 Jan 28;16(1):R28. PMID: 24467809.
Tariq Ezaz, et al. Sequence and gene content of a large fragment of a lizard sex chromosome and evaluation of candidate sex differentiating gene R-spondin 1. BMC Genomics. 2013 Dec 17;14:899. PMID: 24344927.

Eukaryotic cell lines

Policy information about cell lines and Sex and Gender in Research

Cell line source(s)

The following cell lines (and their respective catalog numbers) were from American Tissue Type Collection (ATCC; Manassas, VA): A375 (#CRL-1619) and HEK293T (#CRL-3216).
The following cell lines (and their respective catalog numbers) were from Rockland Immunochemicals (Limerick, PA): WM983A (WM983A-01-0001); WM983B (WM983B-01-0001); WM1366 (WM1366-01-0001); WM115 (WM115-01-0001); WM266-4 (WM266-4-01-0001); WM164 (WM164-01-0001); WM793 (WM793-01-0001); and LU1205 (1205LU-01-0001).
The SM1 murine melanoma cell line was obtained from the Smalley laboratory; SKMEL19 and Meljuso cell lines were obtained from the Karreth laboratory; the IPC298 cell line was obtained from the Tsai laboratory, and the LNCaP human prostate cancer cell line was obtained from the Wang laboratory at Moffitt Cancer Center & Research Institute.

Authentication

The identities of all cell lines (human and mouse) in the Lau Laboratory were initially verified as of December 2019 by short tandem repeat (STR)-based authentication "CellCheck" services provided through IDEXX BioResearch (idexxbioresearch.com). Repeat authentication of cell identities were performed annually on routinely used and newly acquired cell lines. The most

	recent round of authentication was performed as of October 2020. All rounds of authentication have confirmed the proper identities of all cell lines in the Lau laboratory (used in this study).
Mycoplasma contamination	All cells in this study were confirmed to be mycoplasma-free before experiments using the InvivoGen PlasmoTest Mycoplasma Detection Kit.
Commonly misidentified lines (See ICLAC register)	Our authentication validated that the LU1205 cells (a commonly misidentified cell line) used in this study are pure cultures without contaminant lines.

Animals and other research organisms

Policy information about studies involving animals; ARRIVE guidelines recommended for reporting animal research, and Sex and Gender in Research

Laboratory animals	Four-to-six-week-old male C57BL6 mice were purchased from Charles Rivers Laboratories.<br>Four-to-six-week-old male NSG mice were obtained from the Jackson Laboratories.<br>All mice were housed in the Vincent A. Stabile Research Building animal facility at the Moffitt Cancer Center, in rooms on a standard 12-h–12-h light cycle, with a temperature range of 68–72 °F and humidity range of 30–70%.
Wild animals	This study did not involve wild animals.
Reporting on sex	We only used male mice in this paper, as our study primarily focused on delineating the male sex hormone (androgen)-associated poor disease outcomes in male melanomas. To validate the signaling mechanisms that we demonstrated in vitro using a male patient-derived melanoma cell line, it would be more comparable to include male mice. As our study supports the administration of AR inhibitors (ARi) as a potential therapeutic strategy for treating male melanomas, thus male mice were selected in our study to test the effect of ARi on suppressing melanoma progression in preclinical models.
Field-collected samples	This study did not involve field-collected samples.
Ethics oversight	Our research complies with all relevant ethical regulations. All animal experiments were performed in accordance with an Institutional Animal Care and Use Committee protocol (IACUC protocol, #IS00010075) approved by the University of South Florida.

Note that full information on the approval of the study protocol must also be provided in the manuscript.

Plants

Seed stocks	N/A
Novel plant genotypes	N/A
Authentication	N/A
